# Effects of Labeling and Group Category of Evaluators on Evaluations of Aggression

**DOI:** 10.1371/journal.pone.0144384

**Published:** 2015-12-08

**Authors:** Tsukasa Teraguchi, Naoki Kugihara

**Affiliations:** 1 Graduate School of Human Sciences, Osaka University, Suita, Japan; 2 Japan Society for the Promotion of Science, Tokyo, Japan; Mälardalen University, SWEDEN

## Abstract

This study investigated whether the effect of labeling on people’s evaluation of aggression varies according to the group category of the evaluators (i.e., whether they are ingroup members or third parties). Two labeling strategies—the negative labeling of victims (NL strategy) and the positive labeling of aggressors (PL strategy)–were adopted. We conducted an experiment using the hot sauce paradigm, as a way to assess aggressive intent that includes behavioral measures of evaluations. The results suggested that the NL strategy causes ingroup members to evaluate aggression in a more positive light, while the PL strategy has the same effect but on third parties instead. Thus, labeling strategies may increase the severity of aggressors’ reaction and could also be a factor that can escalate a war or conflict.

## Introduction

Labeling involves describing an object in a single word or phrase. Typical examples are as follows: adults call a youth who causes trouble a “troublemaker”; people call a criminal “abnormal”; Barack Obama said “justice” about that Osama bin Laden was killed [1–2]. People have a tendency to use labeling. Since the 1960s, many sociologists and criminologists have studied labeling, and research indicates that labeling might alter the cognitions and behaviors of the persons who are labeled as well as others into the shared perceptions and judgments that the label represents [3–7].and their observers to the shared ones which label represents [3–7]. As such, labeling is a strategy that is often used in the context of legitimizing aggression. For example, Haritos-Fatouros [8] has demonstrated that calling a victim a “worm” causes people to perceive that they are allowed to commit brutal acts against that person. During the conflict between the Tutsi and Hutu in Africa, the Hutu used labels such as “cockroaches” to refer to the Tutsis [9], and there are indications that describing victims as vermin or insects causes mass murder to be perceived as a moral imperative [10]. Furthermore, qualitative study with Palestinian youth about anti-Israeli resistance movements [11] noted that the resistance organizations named themselves “freedom fighters,” and the authors suggested that this label was a claim for the just cause of Palestinian resistance organizations. Empirical research has suggested that in a setting where aggression is predicted, the more aggressive intent the aggressor has, the higher is the likelihood that they will choose a team name that is better than the name of the opponent’s team [12]. Thus, there are two labeling strategies used by aggressors: negative and positive. The one is negative labeling (NL)strategy is when aggressors use negative words such as “cockroaches” and “worm” to describe the victim of aggression or their actions. The intention of this strategy is to demean the victim of aggression. The positive labeling (PL) strategy is when aggressors use positive words to describe themselves or their actions, such as “justice” and “freedom fighters.” Here, the intention is to elevate the aggressor.

Why do aggressors use labeling strategies? According to the extended model of goal-directed behavior [13–14], inference of the reaction of observers to one’s aggressive behavior may influence one’s level of aggression. Thus, aggressors estimate how others will react to their aggressive behavior. In this way, whether others will give a positive or negative reaction to the aggressive behavior is an important factor in acting aggressive [15–16]. To legitimize their behavior, the aggressor may use a variety of strategies. The strategy that will be used at the lowest cost is a labeling strategy. However, few studies have tested whether labeling strategies can actually legitimize aggressive behavior. Research has demonstrated that in terms of the aggressor’s cognitions, dehumanizing labeling of the victim reduces empathy toward the victim [17], and that people become less critical of punishment by electric shock when a dehumanizing, rather than humanizing, label is given to the punished [18]. Regarding the cognitions of third parties, Teraguchi & Kugihara [19] conducted a study using vignettes, and demonstrated that when the victim was labeled as “evil” or the aggressor as “just,” the latter label lowered the positive evaluation of the aggressor in a setting where the aggressor was engaged in one-sided aggression.

Therefore, the purpose of this study was to test in an experimental setting whether the labeling strategy (NL or PL) would change the evaluation of aggressors by others. The NL strategy can be framed as a kind of verbal aggression that degrades the victim; thus, rather than legitimizing aggression, it may produce a negative evaluation by others. If so, the aggressor would not benefit from using this strategy.

For whom is the labeling strategy effective? In a typical situation where aggressive behaviors occur, there are often third parties as well as the aggressors, victims and those involved [20–23]. A third party refers to a person who is neither the aggressor, the victim, nor ingroup members. In addition, third parties have a tendency to intervene in aggression and conflict [20, 22–23]. We can infer that the preference for the NL strategy can differ depending on the group category of evaluators, the degree of the support for aggression by others. Davies, Kahn, & Hutchinson [24] presented American university students with an article on the subject of the Iraq War, which described Iraqi resistance forces fighting against the American army. To control for labeling, they prepared one article that gave the Iraqi opposition forces the positive label of “resistance fighters,” and the other, the negative label of “guerillas”. After reading the article, the students measured the degree to which they supported America’s invasion of Iraq and the degree to which they supported the Iraqi resistance forces. The results showed that students who supported the invasion of Iraq were more supportive of the invasion and less positive toward the resistance forces when they were presented with the article with the label of “guerillas”. In addition, participants who did not support the invasion of Iraq showed no difference in the support of the Iraqi resistance forces between the “resistance fighters” and “guerillas”. Furthermore, according to a study focused on elections [25], candidates who conducted a negative campaign that degraded the opposing candidate were evaluated positively by supporters of their party but negatively by non-supporters. On the other hand, positive campaigns that appealed to the candidates themselves were evaluated positively by both supporters and non-supporters of the party.

Based on the aforementioned studies, use of the NL strategy will elevate an aggressor’s evaluation by members of the aggressor’s ingroup, who are more likely to support the aggressor, but will cause a negative evaluation of the aggressor by third parties, who are less likely to be supportive of the aggressor. Furthermore, the PL strategy, which is uninfluenced by support toward the aggressor, will cause a more positive evaluation regardless of group category. This led us to form the following four hypotheses:

Hypothesis 1a: The NL strategy will cause an aggressor’s ingroup members to evaluate the aggression more positively.
Hypothesis 1b: The NL strategy will cause third parties to evaluate the aggression more negatively.
Hypothesis 2a: The PL strategy will cause an aggressor’s ingroup members to evaluate the aggression more positively.
Hypothesis 2b: The PL strategy will cause third parties to evaluate aggression more positively.

## Materials and Method

### Experimental design

The experiment had a 2 (group category: ingroup and third party) × 3 (labeling: control [CL], negative label of victim [NL], and positive label of aggressor [PL]) factorial design.

### Participants

The participants were 75 (43 male and 32 female) students attending a university in Japan (age: *M* = 19.11, *SD* = 1.06). This sample size was chosen by design, because a previous study [12] suggested that the effect size of the interaction between the degree of the support for aggression and the labeling strategy was large. According to our calculations based on a power = .80, 64 participants was the minimum required sample size for this experiment.

### Ethics statement

Participants gave written consent to take part in this study, and the recruitment and study procedures conformed to the requirements of the 2008 Helsinki Declaration. The Ethics Committee of the Department of Human Sciences, Osaka University specifically approved this study.

### Hot sauce paradigm

This study used the hot sauce paradigm [26] as an indicator of punishment behavior toward the aggressor. It is difficult to measure aggressive behavioral indicators due to ethical issues, and recently there have been many studies that used the vignette method, including questionnaire surveys or distribution games. However, it is difficult to avoid social desirability with these methods, and there is little sense of reality toward the aggression. The hot sauce paradigm, which is used to measure aggressive intent, has been proposed as a method to avoid these problems. In this paradigm, the true intent of the experiment is hidden from the participant, who is asked to add a spicy (e.g., chili) sauce to a food or drink that targets (i.e., victims) will consume. If the participant has aggressive intent or a negative evaluation of the target, they will be less resistant to having the target eat the sauce, and will, in fact, attempt to make the target eat more of the sauce. In other words, the volume of added sauce demonstrates a negative reaction to the target. This method makes it difficult for participants ascertain the experiment’s intent, and it has a higher sense of reality than vignettes do, since the participant can easily infer the harm to the opponent. This paradigm is valued for these reasons, and its validity has been tested in a number of studies [27–30].

In order to make the sauce appear spicy, this study used a mixture of 150g of chili sauce (sweet hot sauce, White Elephant, Co.) and 6g of pepper powder (Kimchi pepper, CA Spice, Ltd.).

### Procedure

**Fig 1:**
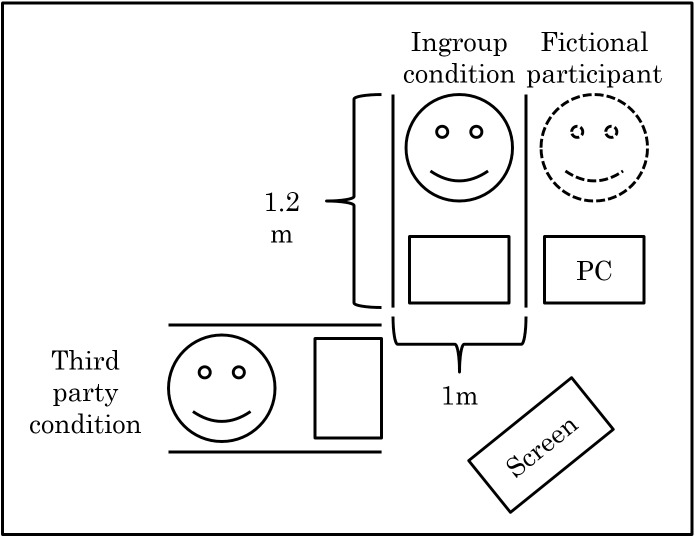
The layout of the laboratory room.

The experiment was conducted in the following environment (Fig 1), with participants taking part in pairs. The participants were not able to see one another, and a personal computer (PC) that they also could not see from their position was set up on its own in the empty seat (right-hand seat in the diagram). However, participants were told that this experiment was conducted in groups of six, with three in their room and the other three in the neighboring room. In addition, the aggressor was presented with a setting in which the victim was doing arithmetic, and the aggressor could play an unpleasant noise to disturb this activity.

The participants were told that the experiment was focused on arithmetic performance and auditory stimulation. First, in order to control for the participants’ group category, those in the ingroup condition were told that they belonged to the same team as the aggressor (fictional participant), and that their team would be competing against another team of two participants in the other room to complete arithmetic problems. Furthermore, they were told that the aggressor would perform an arithmetic problem in the first round, and that they themselves would perform a different arithmetic problem in the second round, with the reward being shared with teammates and the amount changing based on the comparison of the two teams’ scores. Meanwhile, participants in the third party condition were told that were observers; therefore, their reward would not change based on the results of the team that was in the same room as the ingroup condition participant.

Further, participants were told that they could play three kinds of unpleasant noises—quiet, medium, and loud—during the opponent’s calculations by pressing a PC key, and that they could infer the intensity of the unpleasant noises on an audiograph displayed on the screen in front of them. The participants confirmed that a small waveform was displayed on screen for a quiet sound, and a large waveform for a loud sound, and they were told that they would not hear the noise during the actual experiment, but only see the waveform. In fact, the waveforms displayed on screen were all videos produced in advance. Participants were also told that this noise was “extremely effective for disturbing thought,” and that it was “harmful to mental health, and causes pain in the ears if prolonged, so please do not play it for long periods of time.” To ensure that the participants were focused on the screen, they were asked to record on a sheet of paper the degree of the noise that was playing during the task, using a 7-point Likert scale for frequency (1: infrequently; 7: frequently) and length of play (1: short; 7: long).

In order to test the labeling effect, team names were manipulated, with participants being told, “For the sake of convenience in the experiment, team names will be used.” In the PL condition, participants were informed that the aggressor named their own team “Team Anpanman” (a Japanese anime hero), and the victim named their own team “Team A.” In the NL condition, participants were told that the aggressor named the victim’s team “Team Scum”, and the victim named the aggressor’s team “Team A”. In the CL condition, participants were told that the experimenter named the aggressor’s team “Team A”, and the victim’s team “Team B.” The label “Anpanman” appeared often in preliminary research that involved free description of words related to justice, while “scum” is a negative label that has been used in preceding studies [17, 31].

A pretest was conducted before beginning the experiment, and participants were told that the aggressor’s and victim’s arithmetic abilities were equal (131 points vs. 133 points). Therefore, during the experiment, the participant’s team solved the arithmetic problem first, and they were told that in the meantime, the team in the other experiment room would be able to disturb them by playing an unpleasant sound. Next, a video of a small quantity of the disturbing sound (composed of 11.2 seconds of quiet, 15.1 seconds of medium, and 0.9 seconds of loud sound levels) was played on screen for 3 minutes, after which the problem papers were collected. The participants were then asked to record on their sheet the degree to which the team in the other experiment room played the unpleasant noise, and were orally told that they scored 122 points, which was not much different from the pretest score. Next, the participants were told that the team in the other experiment room would be solving the arithmetic problems, and that the participant’s team could disturb them by playing an unpleasant noise. After this, a video of a small quantity of the disturbing sound (composed of 1.1 seconds of quiet sound, 14.1 seconds of medium, and 52.0 seconds of loud sound levels) was played on screen for 3 minutes, after which participants were asked to record on their sheet the degree to which their team played the unpleasant sound. Then, in order to demonstrate that the victims had received a higher quantity of harm, they were told “The other team scored 91 points, so your own team has about a 50% lead.”

After the presentation of the disturbing noise (aggression) was finished, the participants evaluated the aggressor using the hot sauce paradigm. Specifically, the aggressor told the participant that they were continuing on to participate in an experiment on arithmetic performance and taste stimulation, and asked the participant to make a cup of tea mixed with chili sauce, which would be the experimental stimulus. The participant was given a transparent cup (220 cc volume) containing the aforementioned 150g of chili sauce, an empty paper cup (205 cc volume), and a spoon to transfer the sauce from one cup to another. The participant was asked to fill the paper cup with as much sauce as they wanted. Furthermore, in order to demonstrate the evaluator category, the aggressor’s team name was written on a sticky note attached to the paper cup.

Following completion of the hot sauce paradigm, the participants responded to a questionnaire and were debriefed, then the experiment was concluded.

### Measured variables

1) Aggressor/Victim evaluation (aggression evaluation): Participants were asked for their impression of the aggressor (α = .91) and the victim (α = .94), using six items (“aggravating,” “untrustworthy,” “disgusting,” “pitiful,” “irritating,” and “dislikeable”) from the Negative Interpersonal Scale’s [32] “hatred” factor, which are rated on a 7-point Likert scale (1: strongly disagree; 7: strongly agree). Further, for the analysis, all items were reverse scored, so that a lower score indicated a more negative evaluation of the aggressor and victim. 2) Quantity of sauce (behavioral responses): The quantity of chili sauce added by the participant was measured along with the paper cup, and the value (minus the 4.72 g weight of the paper cup) was used as the index. 3) Awareness of harm quantity (manipulation check): In order to check that there was a difference in the use of the disturbing sound between the teams, participants were asked to rate their response to the item, “How much pain do you think each team received from this disturbing sound”, on a 9-point Likert scale (1: no pain at all; 9: extreme pain) for both the aggressor and victim’s teams.

## Results

### Manipulation check

Three subjects who realized the intent of the experimental operation were excluded from analysis, so the data of 71 (40 male, 31 female) student were used. To check that the participants were aware that the aggressor was behaving more aggressively than the victim was, a paired *t* test was run with the variables of awareness of harm quantity received by the aggressor’s, compared to the victim’s, team. Results showed that the awareness of harm quantity received by the victim’s team (*M* = 7.71, *SD* = 1.08) was significantly higher (*t* (70) = 16.17, *p* < .001) than awareness of harm quantity received by the aggressor’s team (*M* = 4.15, *SD* = 1.51).

### Cognitive evaluation of and behavioral response to aggressive behavior

First, to test whether the effect of labeling differed depending on the evaluator’s group category, a 2 (group category: ingroup, third party) × 3 (labeling condition: CL, NL, PL) two-way analysis of variance (ANOVA) was conducted. The dependent variable was the score resulting from subtracting victim evaluation from aggressor evaluation, where a higher score meant that the participant evaluated the aggressor more negatively than they did the victim.

**Fig 2:**
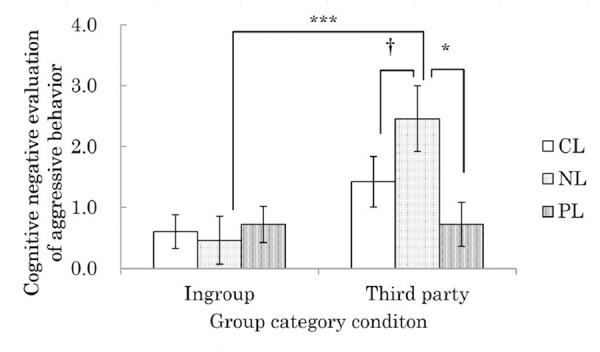
Effects of group category and labeling on the cognitive negative evaluation of aggressive behavior.

The results showed a significant interaction effect of group category and labeling (*F* (2, 65) = 3.19, *p* < .05, partial eta-squared (η^2^) = .09). Subgroup analysis also revealed a simple main effect of group category in the NL condition (*F* (1, 65) = 12.38, *p* < .001, partial η^2^ = .16), in which negative evaluation was higher in the third party condition (*M* = 2.46, *SD* = 1.88) than the ingroup condition (*M* = 0.46, *SD* = 1.29). Further, there was a simple main effect of labeling in the third party condition (*F* (2, 65) = 4.96, *p* < .01, partial η^2^ = .13), and upon conducting multiple comparisons with the Shaffer method, the negative evaluation was higher in the NL condition in either than the CL condition (*M* = 1.42, *SD* = 1.50) or the PL condition (*M* = 0.72, *SD* = 1.26) (CL vs. NL: *t* (65) = 1.90, *p* < .06, *r* = .23; PL vs NL: *t* (65) = 3.13, *p* < .01, *r* = .36; Fig 2; Hypothesis 1b).

Based on these results, the cognitive index only supported Hypothesis 1b, in that the NL strategy caused third parties, compared to ingroup members, to evaluate the aggression more negatively.

**Fig 3:**
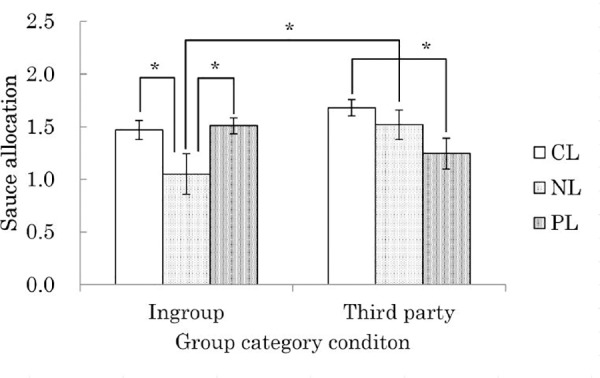
Effects of group category and labeling in the hot sauce paradigm.

Next, a 2 (group category: ingroup, third party) × 3 (labeling: CL, NL, PL) two-way ANOVA was conducted with the quantity of sauce (logarithmically transformed), to see if a similar result could be seen in the behavioral index. The results demonstrated a significant interaction effect (*F* (2, 64) = 4.34, *p* < .05, partial η^2^ = .12). In addition, subgroup analysis demonstrated a simple main effect of group category in the NL condition (*F* (1, 64) = 6.90, *p* < .05, partial η^2^ = .10), with the quantity of sauce being less for the ingroup condition (*M* = 1.05, *SD* = 0.64; not transformed *M* = 22.72, *SD* = 25.48) compared to the third party condition (*M* = 1.52, *SD* = 0.49; not transformed *M* = 48.90, *SD* = 40.77). Furthermore, a simple main effect of labeling was found for both the ingroup condition (*F* (2, 64) = 3.99, *p* < .05, partial η^2^ = .11) and the third party condition (*F* (2, 64) = 3.11, *p* < .06, partial η^2^ = .09). Upon conducting multiple comparison with the Shaffer method, the quantity of sauce for the ingroup condition was found to be lower in the NL condition (*M* = 1.05, *SD* = 0.64; not transformed *M* = 22.72, *SD* = 25.48) than in either the CL condition (*M* = 1.47, *SD* = 0.30; not transformed *M* = 34.92, *SD* = 22.27) or the PL condition (*M* = 1.51, *SD* = 0.26; not transformed *M* = 37.24, *SD* = 24.29) (CL vs. NL: *t* (64) = 2.31, *p* < .05, *r* = .28; PL vs. NL: *t* (64) = 2.58, *p* < .05, *r* = .31; Hypothesis 1a). Further, the quantity of sauce for the third party condition was lower in the PL condition (*M* = 1.25, *SD* = 0.49; not transformed *M* = 27.08, *SD* = 25.12) than the CL condition (*M* = 1.68, *SD* = 0.28; not transformed *M* = 57.07, *SD* = 39.31) (*t* (64) = 2.48, *p* < .05, *r* = .30; Fig 3; Hypothesis 2b).

These results demonstrate that the behavioral responses supported Hypothesis 1a, that the NL strategy would cause the aggressor’s ingroup members to evaluate the aggression more positively, and Hypothesis 2b, that the PL strategy would cause third parties to evaluate the aggression more positively.

## Discussion

The purpose of this study was to investigate whether the labeling strategy (negative labeling of victim, or positive labeling of aggressor) affects the evaluation of aggressive behavior, and whether this effect differs based on the evaluator’s group category (aggressor’s ingroup member, third party).

The results demonstrated that a third party’s cognitive evaluation of aggressive behavior is more negative when the aggressor uses the NL, compared to PL, strategy. In addition, the evaluation was more negative in the third party than in the control condition. Thus, the results of the cognitive index supported Hypothesis 1b. Furthermore, since cognitive evaluation by ingroup members was more positive than that by third parties when using the NL strategy, the Hypothesis 1a was also supported; however, there was no decline in negative evaluation by ingroup members. These results showed that the labeling strategy is not enough to cause a positive change in the cognitive evaluation of aggressive behavior.

However, this study also measured behavioral reactions to the aggressor using the hot sauce paradigm. The results showed that ingroup members gave less sauce to aggressors who used negative labeling of the victim than did those in the control condition or aggressors that used positive labeling of themselves (Hypothesis 1a). On the other hand, those in the third party, compared to control, condition gave less sauce to aggressors who used the PL strategy (Hypothesis 2b). Thus, the aggressor’s ingroup members had a less negative behavioral reaction to aggressors that used the NL strategy, and third parties had a less negative reaction to aggressors who used the PL strategy. Furthermore, the aggressors’ ingroup members gave less sauce to the aggressors that used the NL strategy than third parties did. This suggests that the NL strategy was evaluated relatively more negatively by third parties, which supported Hypothesis 1b.

Integrating these results, it appears that use of the NL strategy against victims diminishes the negative reaction toward the aggressor by the aggressor’s ingroup members, but raises negative cognitions to aggressor among third parties. In contrast, the PL strategy decreased negative reactions of third parties. These results support Hypothesis 1a, 1b, and 2b.

The results of this study are consistent with those of preceding research [24–25]. Davies et al. [24] found that when a nation gives an enemy group the negative label of “guerilla”, the enemy group is more likely to be evaluated negatively by people who support the aggressive behavior of their own country (i.e., the Iraq War). Davies et al. [24] interpreted this finding to mean that the effects of labeling are derived from motivation, such as degrading the enemy group. The results of this study are consistent with that interpretation; in other words, since third parties are not involved with the aggressive behavior, their motivation to degrade the victim is lower than that of the aggressor’s ingroup members. Carrao & Castelli [25] also showed that the effects of a negative campaign on voting behavior differ based on whether the voter supports the candidate’s party. The overall conclusion was that the degree of support for the aggressor’s group and animosity toward the victim’s group has an effect of labeling on evaluators’ responses.

However, the results of this study did not support Hypothesis 2a, that the PL strategy would cause the aggressor’s ingroup members to evaluate the aggression more positively. This may be because of the weaker influence of positive, compared to negative, information. According to Ito, Larsen, Smith, & Cacioppo [33], many studies [34–36] have suggested that positive information has less of an impact than negative information does (i.e., negativity bias), and studies of the brain’s responses also show a smaller effect of positive information. Furthermore, the aggressor’s ingroup members, when required to make a judgment of the aggressor in the group they are affiliated with, may have a large amount of detailed information about the aggressor in comparison with third parties. Crawford, Sherman, & Hamilton [37] have suggested that cognitions about ingroup members are less easily changed than those of outgroup members. According to the above studies, the positive labeling (PL strategy) would be less effective on ingroup than third party members; thus, there is a need to study the use of more arousing positive labeling.

Furthermore, this study demonstrated that positive labeling did not induce positive ratings of aggressive behavior when compared with the control condition. A similar result was found by Carrao & Castelli [25], who only observed a positive effect of a negative campaign in regard to implicit indices (i.e., Implicit Association Test; [38]). Conscious ratings may be strongly influenced by social desirability; in contrast, this influence is weaker in behavioral responses, which suggests that behavioral reactions can be manipulated by labeling strategies.

Based on the present findings, we argue that labeling strategies are one factor that can contribute to the escalation of war and conflict. Moreover, labels such as “justice” and “cockroaches” have been used by aggressors throughout history [2, 8–11]. We found that these strategies not only affect the aggressor’s ingroup members’ behavior, but also third parties in terms of a more positive reaction. Given the aggressors’ decision to engage in aggressive behavior will be based on the reaction of others [13–16], aggressive behavior will increase with the use of labeling strategies, even when there are others who can intervene in the conflict. It is therefore possible that a single word, such as “justice,” “cockroaches,” or “worm,” can cause endless wars. As such, labeling strategies should be considered as dangerous.

However, while the present study found that a difference in the effect of the labeling strategy arises due to the evaluator’s group category, neither the process nor the mechanism by which this happens was uncovered. Future research should clarify what differences occur in the effect of the labeling process based on differences in group category. By doing so, it will be possible to intervene effectively with aggressor’s ingroup members or third parties to avoid their giving a positive reaction to unreasonable aggression in a situation in which labeling is used.

## Supporting Information

S1 Dataset Dataset. (CSV)
